# Using artificial intelligence (CognoSpeak™) in memory assessments: a GP interview study

**DOI:** 10.3399/BJGPO.2025.0098

**Published:** 2026-02-25

**Authors:** Caitlin H Illingworth, Florence Mutlow, Lewis Roberts, Theocharis Stavroulakis, Daniel J Blackburn, Jon M Dickson

**Affiliations:** 1 Division of Neuroscience, School of Medicine and Population Health, Sheffield Institute for Translational Neuroscience (SITRaN), The University of Sheffield, Sheffield, UK; 2 Sheffield Teaching Hospitals NHS Foundation Trust, Sheffield, UK; 3 Sheffield Centre for Health and Related Research (SCHARR), School of Medicine and Population Health, Sheffield, UK

**Keywords:** dementia, information technology, family medicine, artificial intelligence

## Abstract

**Background:**

The memory assessment pathway (MAP) for people with subjective memory deficits (dementia, mild cognitive impairment, and other diagnoses) is under huge strain and new diagnostic technologies have been identified as a high priority for research.

**Aim:**

To investigate the views of GPs on the MAP, and on how an artificial intelligence tool (CognoSpeak™) could be implemented.

**Design & setting:**

Qualitative interview study in a large region of the NHS (South Yorkshire) in England.

**Method:**

Eighteen GPs were recruited using convenience sampling to undertake semi-structured interviews, which were analysed using reflexive thematic analysis (demographic data were monitored to ensure diversity).

**Results:**

GPs think that the MAP has system-wide problems, and that GPs are overworked yet underutilised. They highlighted assessment and referral dilemmas, and the perspectives of patients and families. When asked about implementation of CognoSpeak™, they gave their thoughts on the optimal sites of implementation. The GPs also highlighted barriers and difficulties, as well as the opportunities and benefits, and they made proposals for the future development of CognoSpeak™.

**Conclusion:**

GPs thought effective implementation of CognoSpeak™ could save time, expedite diagnosis, free-up much needed capacity, and improve the longitudinal assessment of people with mild cognitive impairment. A major concern among GPs was the potential for unintended consequences such as creating additional unfunded work. They also felt it could exacerbate difficulties at the intersections between subjective memory deficits and other factors such as low mood, excess alcohol consumption, learning difficulties, or language and culture. They were concerned about poor access to technology among older and more economically deprived people.

## How this fits in

The memory assessment pathway (MAP) is under huge strain, and this is likely to get worse unless changes are made. New diagnostic technologies have been identified as a high priority in dementia research and in the NHS 10-Year Health Plan. This study presents the views of GPs on how an artificial intelligence tool (CognoSpeak™) could be implemented in the MAP. GPs expressed positive views about the potential benefits but warned of unintended consequences and highlighted difficulties.

## Introduction

The number of people with dementia in the UK is projected to almost double to 1.6 million by 2040^1^ and the number of people experiencing cognitive difficulties that are not caused by dementia is also rising sharply.^2^ Primary care is where most people with memory problems are initially assessed and therefore where the memory assessment pathway (MAP) usually begins. GPs are required to refer those patients they suspect may have dementia,^3^ make alternative diagnoses, reassure patients if there are no signs of serious illness, and promote brain health. During this process, GPs must navigate a complex range of clinical, ethical, and practical issues.

Waiting times for memory clinics in the UK’s NHS are up to 2 years. The stakes of delayed diagnosis are growing as new disease-modifying treatments for Alzheimer’s disease (the most common form of dementia), which are most effective when administered early in the disease,^4,5^ come closer to being approved for use. Primary care and secondary care, which work collaboratively in the assessment of memory problems, must adapt if they are to meet the sharply increasing demand that they face.

New diagnostic technologies have been identified as a high priority in dementia research, and in the NHS 10-Year Health Plan.^6^ Computer-based assessments that use artificial intelligence have the potential to improve the efficiency and accuracy of the MAP.^7^ CognoSpeak™is a research tool under development that can be undertaken at home or in a clinical setting. The CognoSpeak™ avatar engages users in conversation. The tool uses artificial intelligence and speech technology to analyse language and speech patterns. CognoSpeak™ has not been implemented yet, but recently published data show that it can distinguish between patients who have dementia from those with other memory complaints, with accuracy comparable with the current manually administered tests.^8^

We aimed to understand the views of GPs on how CognoSpeak™ could potentially be implemented in the NHS. Our recent review highlighted many problematic elements to the current MAP, including significant geographical variation in how services are provided. During implementation it will be important to ensure that CognoSpeak™ does not exacerbate the current problems, and ideally provides solutions, so that it can improve the MAP and does not generate negative unintended consequences. Therefore, we designed a study with the following two aims: (1) to investigate the views of GPs on the current MAP; and (2) to investigate how GPs think CognoSpeak™ could be implemented within the MAP.

## Method

### Study design, setting, recruitment, and sampling

We undertook a cross-sectional qualitative study using semi-structured interviews.^9^ GPs were recruited from practices within the South Yorkshire Integrated Care System boundary, which is the region where CognoSpeak™ is being developed. The study was advertised through formal and informal networks. We used convenience sampling,^10^ but collected and monitored demographic data to ensure that the sample was diverse (see Participants section in the Results). GPs who expressed an interest in taking part were provided with a participant information sheet. Written informed consent was obtained at the beginning of each interview. Based on our prior research experience, we expected that a sample size of 15–20 participants would be sufficient to reach data saturation.^11^ This sample size is similar to recently published primary care dementia studies.^12,13^

### Data collection and management

Each participant undertook a single in-depth online semi-structured interview (reimbursed £80 for their time). The interviewers (FM and CHI) followed a topic guide (supplementary material) using discretion based on the participants' responses. The topic guide was devised by the authors and based on a review of local and national guidelines relevant to the dementia MAP and a review of the published literature.^14^ The interview and topic guide were divided into two halves, the first half was focused on the current MAP and aimed to elicit problematic elements of the pathway and associated processes, the second half was focused on CognoSpeak™ and how it could be implemented in the best possible way despite the problems with the current MAP. Two pilot interviews were conducted to allow refinement of the topic guide; data from the pilots was not included in the final dataset.

Each interview began with an introduction by the researcher summarising the MAP using a flowchart based on our review^14^ and a short video demonstrating CognoSpeak™ (6 minutes duration). Interviews were video recorded using Google Meet, participant demographics were collected using a Google Form, field notes were taken, and all data was transferred to a secure drive at the University of Sheffield. Recordings were transcribed by a professional transcribing service that did not have access to identifiable information. All data were saved using a unique alphanumeric code to protect the participants' anonymity and retained in accordance with the Data Protection Act 1998 and our Data Management Plan. Two researchers (CI and FM) conducted all the interviews, which ended when data saturation was reached.

### Data analysis

The transcripts of all interviews were analysed using reflexive thematic analysis.^15^ The analysis was led by three researchers (CI, JMD, TS) in fortnightly data analysis group (DAG) meetings, plus contributions from the other authors. The initial DAG meetings reviewed a sample of the transcripts to familiarise the researchers with the data and they discussed their preliminary thoughts. After line-by-line reading of all the transcripts, CI generated an initial code framework. Initial themes based on the code framework were discussed in DAG meetings, and they were revised based on DAG discussions with repeated moving back and forth across the whole dataset. NVivo software (version 14) was used to assist the analysis and manage the data.

To ensure methodological rigour and to monitor for data saturation, a process of critical reflection was implemented throughout the data collection phase. This involved iterative review of incoming transcripts enabling the researchers to refine the interview topic guide and explore emerging themes in greater depth. Complementing this, the researchers and the interviewers kept a reflexive diary in which they documented reflections on their own epistemological and methodological stance, their motives for conducting the study, and the influence of personal factors such as professional background and their relationships with participants. These issues were discussed in DAG meetings, contributing to a robust and transparent analytical process.

### Patient and public involvement and engagement

No patient and public involvement and engagement (PPIE) was undertaken specifically for this GP interview study, but the development of CognoSpeak™ has been informed by several workshops and focus groups with clinicians and patients.

## Results

### Participants

We interviewed 18 GPs, the male-to-female ratio was 10:8, their mean age was 44.1 years (standard deviation [SD] 9.1 years), and they had been qualified for a mean of 13.4 years (SD 10.4 years). They undertook a mean of 5.2 (SD 1.4) general practice clinics per week and they all made regular referrals to memory clinics: 1–5 per year (*n* = 4), 6–10 per year (*n* = 4), 11–15 per year (*n* = 8), >20 (*n* = 2). The percentage of participants who identified as part of a minority ethnic group was 22% (4/18): mixed ethnic groups (one), Asian British (three). The practices at which the GPs worked included affluent and deprived areas (including Sheffield Deep End practices). The interviews lasted a mean of 51.45 minutes (SD 8.33).

### Themes and sub-themes

**Figure 1.**
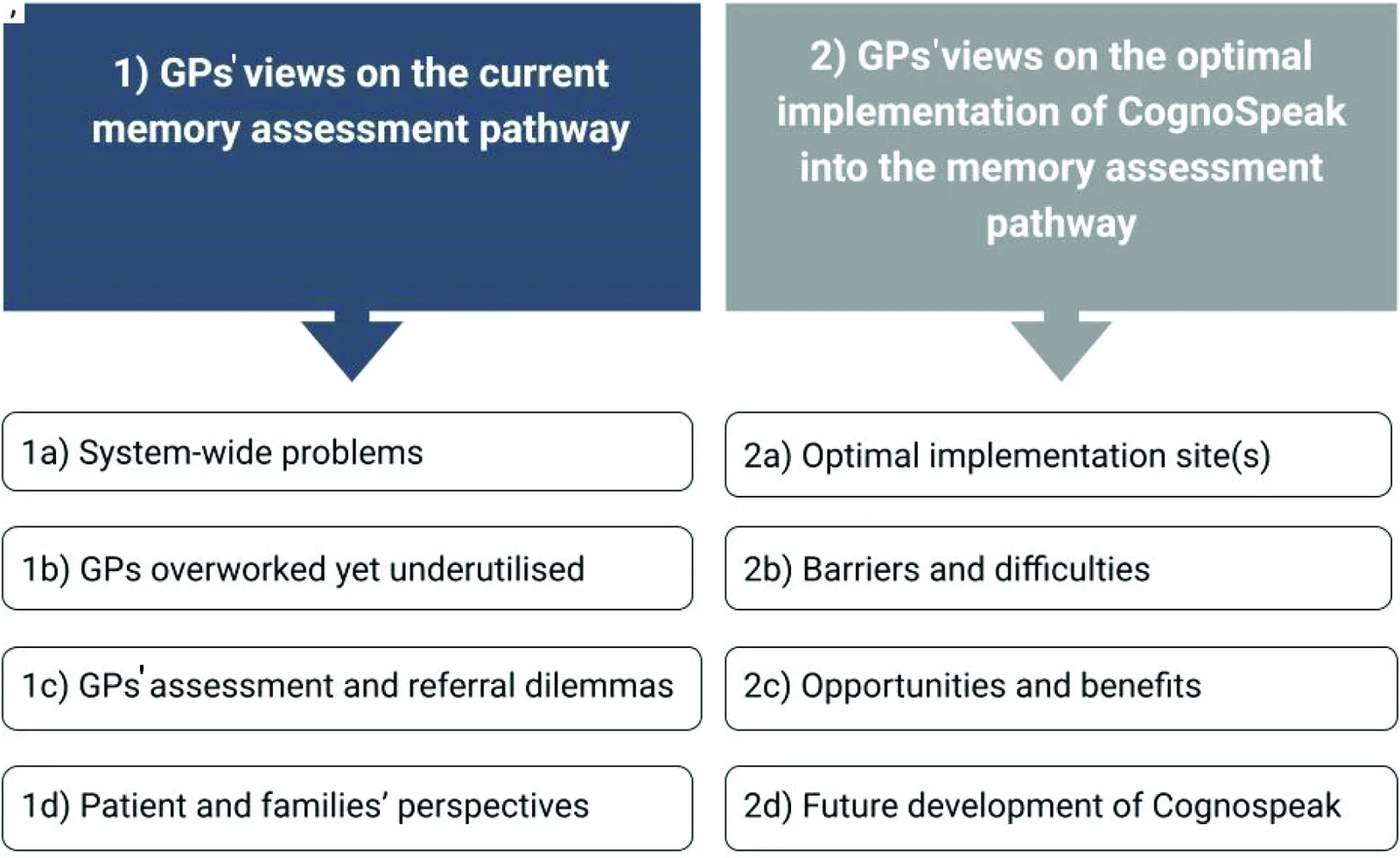
Themes and sub-themes

Findings from the interviews are presented in two main themes according to the two separate but related aims of the study. Within each of the two themes, we identified four sub-themes (see Figure 1).

### Theme 1: GPs’ views on the current memory assessment pathway

#### System-wide problems

GPs spoke about issues within the MAP reflecting wider problems across the NHS, namely lack of funding and lack of resources. GPs spoke about secondary care work being shifted onto primary care without the corresponding funding. Many GPs expressed frustration about the *‘referral hoops you've got to jump through*’ (GP11) for patients to be seen by secondary care describing them as overly rigid and in some cases impractical, for example, rejecting referrals because of co-existing depression or excess alcohol consumption: *‘I think if someone is very rigid about their use of guidelines then potentially they might not use a service when a patient could still benefit from it*’ (GP10).

Some GPs spoke about their experience of having to work around the guidelines to get their patient to secondary care: *‘it’s a bit of game-playing*’ (GP16). They also said that the interface between primary care and secondary care did not allow good shared care and communication especially while waiting for a diagnosis:

*‘... once I’ve kind of referred them I feel like after that, it’s completely out of my hands. There’s no collaboration between primary and secondary care after that point.*’ (GP13)

Despite growing referral numbers, GPs thought that claims of over-referral were wrong:

*‘I’d still be very hesitant about secondary care taking less and less patients … I think secondary care need to be seeing as many if not more.*’ (GP05)

*‘I have a patient that told me about their memory concerns, it sounds like they're relatively mild but significant, still significantly impacting their day-to-day life. It might be quite early memory impairment but it’s definitely different from before, I haven't found an alternative cause. I don't really want anything else in the way of me making a referral.*’ (GP01)

Overwhelmingly, GPs felt that the biggest issue in the MAP was the wait time patients face following a referral to secondary care: *‘people are dying before they get* [there]’ (GP06). This was seen as not only difficult for patients and families but also rendering the memory service ultimately useless:

*‘I think it’s the waiting list, the waiting list cannot; like if it just carries on going up it basically isn’t a service, because by the time they get there they can’t have the medicines or it’s too late or they’ve missed the boat.*’ (GP14)

Many were pessimistic about the possibility of improvement without drastic changes to the whole NHS system, and many seemed resigned to just working through the system already in place: *‘Um* [sighs] *it’s, it’s, it’s OK, it’s OK, it’s what we’ve got*’ (GP10).

#### GPs are overworked yet underutilised

Despite being experienced doctors, often with lifelong relationships with their patients, GPs felt that their ability to make a pragmatic preliminary diagnosis, or in some cases a definitive diagnosis, was underutilised and they disliked the lack of trust in their clinical judgement compared with the *‘tick-box*’ (GP12) referral process:

*‘I think it’s my, my history and conversations with the, the patient that really make my decision ...*’ (GP07)

GPs understood and to some extent agreed with the NICE dementia guideline,^3^ which states that diagnosis should only be made in a specialist memory clinic:

*‘... as GPs we’ve always been told you can’t diagnose dementia.*’ (GP05)

*‘I wouldn’t feel confident to do it based solely on sort of level of assessment that I do in primary care.*’ (GP08)

But GPs spoke about the need for pragmatic diagnoses to be made more readily in primary care when their patient’s neurodegeneration was obvious and had progressed to the extent that waiting to be seen in a specialist clinic came with very few benefits:

*‘... us in primary care are becoming more confident and developing skills to be able to diagnose dementia.*’ (GP15)

GPs reported having very little time available for complex cases of suspected dementia and wasting the available time on unnecessarily complex referral forms, with the to-ing and fro-ing of referral rejections and clarifications. Managing expectations during very long waiting periods for memory clinics is particularly difficult for GPs:

*‘So the initial assessment itself is that, it’s predictable, but kind of then coming back in the interim is, is the additional kind of time burden.’* (GP14)

*‘ ... it just seems like this endless task of them coming back and back and back and back, when you can literally do nothing. Like so it, you just feel so, I don’t know, useless and helpless, and like they get, and the family’ll get more and more frustrated and angry, and it’s all directed at you* [laughs] *often when you’re like, there’s nothing that you can do.*’ (GP14)

#### GPs’ assessment and referral dilemmas

Many GPs spoke about the difficulties they encountered when performing the initial assessment, especially in patients with significant comorbidities:

*‘... it’s very rare to get someone who’s just got a memory impairment, nearly always they’ve got a previous history, and again you’re trying to figure out is this a vascular condition or what is it.*’ (GP11)

Specifically, a few GPs spoke about the difficulty of disentangling the impact of co-occurring mood disorders from a neurodegenerative memory loss:

‘... *is this just anxiety or depression, or just dementia? Cos quite often, patients will have both. And it’s really complex to unpick how much impact each is having.*’ (GP07)

GPs spoke about the anxiety they felt about a *‘wasted referral*’ (GP13) for people who ultimately did not have dementia, and on the other hand the huge impact of a confirmed dementia diagnosis for individuals and their families: *‘I think it is just cos it’s such a life-changing diagnosis, more than most other diagnoses’* (GP14). Some GPs also spoke about the difficulty of managing patients unwilling to proceed with a primary care assessment or a referral for a diagnosis:

*‘I would say it’s often quite a sensitive subject, people are often not completely willing to come in … cos they’re afraid of getting a diagnosis.*’ (GP14)

And that family members often push for an assessment or referral despite their relative’s reluctance, *‘often the, the agenda is being pushed by the family member*’ (GP12).

#### Patients’ and families’ perspectives

Many GPs talked about the difficulties from the perspective of patients and their families:

*‘They know that there are medicines that help prevent, er like slow down the deterioration, and they recognise that they’re missing out on that, which is really challenging.*’ (GP14)

GPs also spoke about the difficulty of families witnessing their loved one deteriorate while waiting to be assessed in a memory clinic, *‘it’s just so sad, um, and it’s just so hard for the families to watch them deteriorate for two years. It’s just a massive ask that, isn’t it, without any help at all*’ (GP14), because access to dementia services is often restricted until there is a formal diagnosis made in a memory clinic.

GPs also spoke about the *‘stigma and a lot of fear*’ (GP12) that some patients and families face when seeking a dementia diagnosis. GPs spoke about the lack of consideration for the additional barriers patients from ethnic minorities can face:

*‘I think it’s that embarrassment and shame in um families in ethnic minorities tends to focus on, um, and it prevents them getting the support or going to their GP*.’ (GP16)

‘... in *my community, dementia isn’t a thing, White people get dementia, Brown people apparently don’t get dementia. So it was almost like, really, our dad’s got dementia? No, they’ve got that wrong, kind of thing*.’ (GP16)

### Theme 2: GPs’ views on the optimal implementation of CognoSpeak™ into the memory assessment pathway

#### Optimal implementation site(s)

Many GPs spoke about the potential for using CognoSpeak™ in primary care with GPs *‘gatekeeping*’ (GP12) access. Some suggested that a CognoSpeak™ assessment could be offered *‘before even seeing the GP*’ (GP12), or during a GP consultation. Other GPs spoke about the potential for incorporating CognoSpeak™ into the current GP assessment process with the help of a healthcare assistant (or other healthcare professional):

*‘... how I could see it working is you do the history and examination. Then you take a — go to reception now and reception will show you how to do* CognoSpeak™. *You do that. Go for your blood test and then come back and we’ll talk about the results and then in that result appointment you would — either reassure them or refer them on.*’ (GP16)

Some GPs felt that CognoSpeak™ would be more appropriately placed in secondary care, *‘when I look at that and think about that, I sort of see far more benefit in secondary care than primary care. Erm, perhaps because I think primary care’s already quite full of these sorts of pre-assessments and things like that*’ (GP03). Or as a *‘pre-screening for the memory clinic*’ (GP03) following a GP referral.

A few GPs spoke about the potential for CognoSpeak™ to be rolled out as an openly available memory screening tool:

*‘I think make it directly accessible for patients so that they can use it without needing to wait for a GP appointment or have a GP refer them*.’ (GP05)

Some GPs saw no place for CognoSpeak™ at all:

*‘Unless … Cognospeak negates the whole need to see a GP, I can't see, I can't see where it’s gonna get implemented.’* (GP09)

#### Barriers and difficulties

While many GPs were open to using a tool such as CognoSpeak™, almost all expressed concerns especially about the possibility of it adding to GPs workload, increasing patient waiting times and other difficulties that may cause patients to *‘drop out of the process and then never end up getting a referral to the service they need*’ (GP08).

The potential for technological barriers excluding patients from completing CognoSpeak™ was a common concern across GPs. Given the lower levels of technological confidence seen in the age group most at risk of developing dementia, there was concern that many patients with memory concerns would be unable to complete CognoSpeak™ without assistance:

*‘I think there are a lot of patients that don’t, especially elder, older patients that don’t, don’t use technology.*’ (GP17)

*‘... you’re gonna create inequalities with people who don’t have access to computers, internet or, you know, maybe even language barriers.*’ (GP06)

GPs spoke about patients’ preference to speak to a person when they have a health concern, and resistance to engaging with online alternatives to traditional clinical appointments, as a reason that many may be unwilling to use CognoSpeak™:

*‘... you’re gonna sit on a computer, I think would be a really hard sell.*’ (GP14)

*‘I just think we just cannot lose completely the human touch on it; like I just think that patients would struggle so much if we say, oh go and speak to this person, and then they get another AI thing and they get another AI thing, I think it’s just a really sad way that it’s going if that’s the case*.’ (GP14)

At the time of the interviews, the proposed threshold for referral in CognoSpeak™ was a probability of dementia of>50% which GPs thought was unacceptable:

*‘Am I missing something, I don't think I am but am I missing something, I'd be interested to know what other people think about it— where they see it in the referral process. And how do they feel about the 50% yes, 50% — it’s like flipping a coin.*’ (GP09)

#### Opportunities and benefits

Many GPs recognised the potential benefits for both patients and clinicians in using tools such as CognoSpeak™ in the MAP. Some saw CognoSpeak™ as a tool to both save GP time and lower anxiety when deciding whether to refer a patient to memory care:

*‘I think, like I mentioned before, it would help with the borderline cases. I already feel quite confident with diagnosis of both ends of that spectrum. But in the — in the unclear cases, I think it would really help*.’ (GP13)

GPs also spoke about the benefit of using CognoSpeak™ to monitor patients discharged from memory service with mild cognitive impairment (MCI):

‘...*in terms of monitoring it’d be quite helpful, particularly if you’re able to get sort of a more standardised score, you know, “oh well you had this assessment a year ago, we’ll repeat it and we’ll see if that standardised score has dropped”, I think that’s quite a useful thing to be able to do and I can certainly see that that would drop down significantly the numbers of those patients who go on to have a re-assessment by memory clinic perhaps unnecessarily*.’ (GP08)

GPs spoke about CognoSpeak™’s potential to help combat the distress that comes with the uncertain and long waits to be seen in secondary care. The potential to provide faster reassurance or confirmation of patients’ concerns and to help families to understand the deterioration of their loved one was described as a large potential benefit for patients and their families to better manage the condition:

*‘So it’s a really good, objective tool, isn’t it, to be able to assess how much worse, not only from the clinician point of view but also from the family’s point of view about the rate of deterioration*.’ (GP16)

#### Future development of CognoSpeak™

While GPs were hopeful for a tool such as CognoSpeak™, they also expressed scepticism and highlighted unanswered questions:

*‘I think it’ll be, it’ll be exciting to see where it goes um, but, as I say, I think it’s, it’s, it, it’s working out how to make it so it’s actually helping streamline a process rather than adding more information and more steps to a process.*’ (GP12)

Most GPs wanted more information on exactly how to implement CognoSpeak™ for their patients, and how to deal with the output of the assessment: *‘the question I'm always thinking is about where is this gonna fit into my referral process, my time pressure*’ (GP09), *‘So um the question again would be if somebody says, I’m worried about my memory, and then the, the screening thing, the* CognoSpeak™ *says, well you don’t meet threshold for referral, where’s that then meant to go?*’ (GP12). Many GPs suggested pilots or tests for CognoSpeak™ to help to establish the optimal method of implementation.

There was also a desire to see more research to determine the accuracy and usability of CognoSpeak™ in a diverse patient population:

*‘Does it work with different languages was another thought I had; … how’s that gonna pick between someone with learning difficulties and somebody who’s just not as bright, who can't name animals quickly and somebody who isn't used to speaking in this way in assessments, how does, is CognoSpeak any good at picking up on that?*’ (GP09)

## Discussion

### Summary

GPs described a failing memory assessment pathway. The mismatch between the number of patients presenting to primary care with a subjective memory deficit and the capacity to safely and accurately assess them results in huge waiting lists for specialist secondary care clinics. GPs felt overworked and underutilised, and patients and families felt distressed and let down.

GPs saw potential benefits of CognoSpeak™ and they proposed implementation in either (1) primary care, (2) secondary care, or (3) as an openly available tool. They thought effective implementation could save time, expedite diagnosis, and free-up much needed capacity, and they could see an important role for CognoSpeak™ in the longitudinal assessment of people with MCI aligning with the vision of increased use of technology in the NHS 10-Year Health Plan.^6^ However, GPs wanted more detailed information and evidence for the benefits of different models of implementation.

Some GPs saw no useful role for CognoSpeak™ and did not think that it should be implemented. A major concern among GPs was the potential for unintended consequences such as additional unfunded workload in primary care ultimately adding to the core problem of lack of capacity. They were concerned about poor access to technology among older people (the key demographic) and economically deprived people. They highlighted the difficulties at the intersections between subjective memory deficits and other factors such as low mood, excess alcohol consumption, learning difficulties, language, and culture, for which the current MAP does not have a satisfactory approach and which CognoSpeak™ may exacerbate.

### Strength and limitations

Programmes of health policy and research often neglect the view of GPs, but this study has elicited the views of GPs from a large NHS region at an early stage, ensuring that their perspective can contribute to future development of CognoSpeak™. CognoSpeak™ is still a research tool and is not available on the NHS, therefore none of our participants had direct experience of using it in clinical practice. They did, however, receive a full briefing on its current specifications, and how patients and clinicians interact with it in its current form.

### Comparison with existing literature

There is a large literature on the MAP, which has been reported elsewhere,^14^ but as far as we are aware this is the only study that has investigated the views of GPs on implementation of an artificial intelligence tool in primary care for assessment of memory problems. In terms of sensitivity and specificity, CognoSpeak™ performs at a similar level to the commonly used Six-item Cognitive Impairment Test (6CIT) for both dementia and MCI.^8,16^ The probability cut-off to be used in practice with CognoSpeak™ is part of ongoing work and the importance of this issue was highlighted by GPs in this study. The ongoing National Institute for Health and Care Research (NIHR) Invention for Innovation (i4i) funded trial will provide more diagnostic accuracy data, and improve usability especially for those from ethnic minorities and for whom English is not their first language. It is hoped that this will lead to a diagnostic technology that can be implemented in the NHS.

### Implications for research and practice

CognoSpeak™ has the potential to reduce waiting lists and improve the MAP both for clinicians, and for patients, reducing stress and inefficiency. The optimal site(s) at which CognoSpeak™ could be implemented is a key consideration and requires further research.

GPs could potentially take on a bigger role in the diagnosis of dementia, which is an argument in favour of implementing CognoSpeak™ in a primary care setting (rather than in secondary care). Such an approach would allow care to be delivered closer to home and by medical generalists who are arguably in the best position to take into account other health conditions relevant to memory complaints and a potential diagnosis of dementia. GPs could potentially be empowered to safely make the diagnosis of dementia especially in those with a high pretest probability such as older people with clear evidence of progressive cognitive and functional decline (and, vice versa, to exclude dementia in young people without evidence of decline). This could become part of the standard skill set of GPs, or perhaps it is better suited to GPs with an extended role (GPwER) with enhanced training. Such a change could be based on traditional memory assessment tools such as 6CIT or using new tools such as CognoSpeak™.

More sophisticated approaches to assessing patients at the intersection between subjective memory complaints and other factors are required. A Bayesian approach using pretest probability based on demographic factors, particularly age, comorbidities, cognitive test results, collateral history, and potentially fluid-based biomarkers, could be developed and integrated into a technology such as CognoSpeak™.

## Data

Data for this study are not publicly available.
